# Comprehensive analysis of the competing endogenous circRNA-lncRNA-miRNA-mRNA network and identification of a novel potential biomarker for hepatocellular carcinoma

**DOI:** 10.18632/aging.203056

**Published:** 2021-05-28

**Authors:** Lu Zhang, Haisu Tao, Jiang Li, Erlei Zhang, Huifang Liang, Bixiang Zhang

**Affiliations:** 1 Hepatic Surgery Center, Tongji Hospital, Tongji Medical College, Huazhong University of Science and Technology, Wuhan 430030, Hubei, China; 2 Hubei Key Laboratory of Hepato-Pancreato-Biliary Diseases, Wuhan 430030, Hubei, China

**Keywords:** HCC, ceRNA network, prognostic signature, DTYMK

## Abstract

Background: The competing endogenous RNAs (ceRNAs) hypothesis has received increasing attention as a novel explanation for tumorigenesis and cancer progression. However, there is still a lack of comprehensive analysis of the circular RNA (circRNA)-long non-coding RNA (lncRNA)-miRNA-mRNA ceRNA network in hepatocellular carcinoma (HCC).

Methods: RNA sequencing data from The Cancer Genome Atlas (TCGA) and Gene Expression Omnibus (GEO) database were employed to identify Differentially Expressed mRNAs (DEmRNAs), DElncRNAs, and DEcircRNAs between HCC and normal tissues. Candidates were identified to construct networks through a comprehensive bioinformatics strategy. A prognostic mRNA signature was established based on data from TCGA database and validated using data from the GEO database. Then, the HCC prognostic circRNA-lncRNA-miRNA-mRNA ceRNA network was established. Finally, the expression and function of an unexplored hub gene, deoxythymidylate kinase (DTYMK), was explored through data mining. The results were examined using clinical samples and *in vitro* experiments.

Results: We constructed a prognostic signature with seven target mRNAs by univariate, lasso and multivariate Cox regression analyses, which yielded 1, 3 and 5-year AUC values of 0.797, 0.733 and 0.721, respectively, indicating its sensitivity and specificity in the prognosis of HCC. Moreover, the prognostic signature could be validated in GSE14520. The prognostic ceRNA network of 21 circRNAs, 15 lncRNAs, 5 miRNAs, and 7 mRNAs was established according to the targeting relationship between 7 hub mRNAs and other RNAs. Our experiment results indicated that the depletion of DTYMK inhibited liver cancer cell proliferation and invasion.

Conclusions: The network revealed in this study may help comprehensively elucidate the ceRNA mechanisms driving HCC, and provide novel candidate biomarkers for evaluating the prognosis of HCC.

## INTRODUCTION

HCC, as the most common type of liver cancer, has become one of the main causes of cancer-related death, and it is also a global health problem that has attracted widespread attention [1, 2]. Due to the lack of obvious clinical manifestations of early HCC, most patients are already in advanced stages when the first symptom appears, and thus they miss the chance of radical resection [3, 4]. Although great progress has been made in the diagnosis and therapeutic techniques of HCC, the recurrence-free survival (RFS) and overall survival (OS) in patients with HCC are still relatively short [5, 6]. In addition, owing to the lack of comprehensive understanding about the complex disease process and molecular interactions in HCC, there is no effective biomarker for prognosis in clinic. Therefore, it is urgent to find potential biomarkers and new targets to predict the OS and RFS of HCC patients, so as to improve the prognosis and guide individualized treatment.

In 2011, some researchers proposed the ceRNA activity, which can unify the transcriptome and form a large-scale regulatory RNA network. They described a complicated post-transcriptional regulatory network, in which circRNAs, lncRNAs and other RNAs can compete with miRNAs and act as natural miRNA sponges by virtue of sharing no less than one miRNA response element (MRE). Since these noncoding RNAs (ncRNAs), and protein-coding mRNAs can combine with miRNAs through MRE, they can compete with miRNAs and participate in the regulation of this complex network [7, 8]. Many studies have found that ceRNA regulation can have an important impact on the occurrence and development of HCC [9–11]. Thus, these networks could be used to gain insight into complex gene interactions and identify potential biomarkers to diagnose and treat HCC.

The RNA sequencing data of TCGA or GEO database can provide circRNA, miRNA and mRNA data of various cancers, which can be an excellent resource for data mining and biological discovery [12]. Based on these public databases, integrative ceRNA regulatory networks were constructed to explore more accurate prognostic markers in numerous studies. For HCC, prognostic lncRNA-miRNA-mRNA [4, 8] and circRNA-miRNA-mRNA [13] ceRNA networks have been published. However, there is no research simultaneously including lncRNAs and circRNAs in the ceRNA network of HCC.

In the current research, TCGA and GEO databases are used to identify DEmRNA, DElncRNA and DEcircRNA between HCC and normal tissues, so as to ensure the accuracy and repeatability of the analysis results. The target miRNAs of DEcircRNAs and DElncRNAs were respectively predicted and crossed to obtain common miRNAs and their target mRNAs. Then, a prognostic mRNA signature was established using the data from the TCGA database and successfully validated in GSE14520. The DTYMK hub mRNA in this model was screened for further research and validated as a potential biomarker.

## MATERIALS AND METHODS

### Data retrieval and processing

We downloaded RNA-seq profiles of HCC patients from TCGA database. The circRNA microarray GSE94508 and GSE97332 (GPL19978, gilent-069978 Arraystar Human CircRNA microarray V1) datasets were collected from the GEO dataset. GSE94508 includes 5 adjacent nontumor tissues and 5 tumor tissues. GSE97332 contains 7 adjacent nontumor tissues and 7 tumor tissues. The lncRNA expression profile of GSE138178 comes from the GPL21827 platform (Agilent-079487 Arraystar human LncRNA microarray V4), which contains 49 pairs of HCC tissue and adjacent non-tumor tissue samples. The “limma” package in R software was applied to screen DEcircRNAs, DElncRNAs and DEmiRNAs between HCC samples and adjacent normal samples. We identified the significant DEcircRNA in GSE94508 and GSE97332 profiles (p <0.01 and |LogFC|> 2). The thresholds of |LogFC| >1 and p < 0.05 were used to screen the significantly DEmRNAs and DElncRNAs on the TCGA-LIHC database. Then, the same standard was applied to analyze lncRNA data from GEO microassay GSE138178.

### Prediction of targeting relationship

The CircInteractome database was used to predict the DEcircRNAs targeting miRNAs (circ-pre-miRNAs) [14]. MiRcode database was used to predict the target miRNAs of DElncRNAs. The target mRNAs of miRNAs were obtained from the TargetScan, miRTarBase, and miRDB databases [15–17]. In order to improve the reliability of the results, we only selected those miRNA-mRNA relationship pairs that overlap in all three databases for further research. These interaction relationship pairs were visualized by the Cytoscape software [18].

### Prognostic model construction

In order to determine the relationship between the patients' mRNA expression and OS, univariate Cox proportional hazard regression analysis, the lasso penalty Cox analysis and multiple Cox regression analysis were carried out, and a prognostic model was constructed. The *survminer* R package was used to calculate the optimal cut-off value, and then the patients were divided into high- and low-risk cohorts. By using *survival* R package, the Kaplan-Meier survival curve (KM curve) could be used to compare prognostic significance.

### Validation of the prognostic model

The prognostic capacity of the risk score was analyzed through univariate and multivariate Cox regression analysis. GSE14520 datasets were downloaded to verify the prognostic characteristics of this signature. Next, by using the KM curve and ROC curve, the predictive value of prognostic gene signature could be tested.

### Gene set enrichment analysis (GSEA)

The gene sets of “h.all.v7.1.symbols.gmt [cancer hallmarks], c5. all. v7.1. symbols. gmt [gene ontology (GO) term] and c6.all.v7.1.symbols.gmt [oncogenic signatures]” from the Molecular Signatures Database were analyzed using the software GSEA 4.0.3 [19]. In addition, HCC samples were divided into high and low DTYMK expression groups according to the median of DTYMK expression value, and analyzed by GSEA. The statistical significance was based on the threshold of P < 0.05 and FDR (false discovery rate) q < 0.05.

### HCCDB, human protein atlas and GEPIA

The HCCDB database (http://lifeome.net/database/hccdb) is an open-access online resource that contains thousands of clinical samples data from multiple HCC datasets [20]. In HCCDB, we analyzed the expression of DTYMK in HCC across multiple datasets. The Human Protein Atlas (HPA) (https://www.proteinatlas.org) is a publicly available interactive website tool that contains gene expression and protein levels data [21]. GEPIA is an interactive web that includes 8,587 normal and 9,736 tumors samples [22]. We used these tools to analyze the protein levels of DTYMK expressed and survival curves, including RFS and OS.

### Sample collection, cell culture, and transfection

The 47 pairs of HCC tissues and paracancer normal tissues were obtained from the Hepatic Surgery Center of Tongji Hospital. This study was approved by the Medical Ethics Committee of Tongji Hospital. MHCC-97H [97H] and HepG2 [G2] were purchased from China Center for Type Culture Collection (CCTCC, China) and cultured in DMEM (Gibico, USA) supplemented with 10% fetal bovine serum (FBS) at 37° C in 5% CO2. Small interfering RNA (siRNA) and the corresponding negative control (siNC) were purchased from Ribobio (Guangzhou, China). 97H and G2 cells were transfected with siDTYMK or siNC by using Lipofectamine 2000 (Thermo Fisher Scientific, USA) following the manufacturer’s protocol.

### Quantitative real-time PCR

Total RNA was extracted from HCC patient tissues and cells using TRIzol Reagent (Life Technologies, USA) and reverse transcription was performed using the PrimeScript® RT reagent Kit (Takara Bio, Japan). Quantitative RT-PCR (qRT-PCR) was performed on a CFX Connect ™ Real-Time PCR Detection System (Bio-Rad, USA) with SYBR Green Supermix kit (Takara Bio, Japan) according to the manufacture’s instruction. The primer sequences were listed as follows:

DTYMK(forward):5′-CCGGTTCCCGGAAAGATCAAC-3′;

DTYMK(reverse):5′- TCCCAGCGATTTGCAGAAAAA-3′.

### Cell proliferation assay and EdU assay

We performed the Cell Counting Kit-8 (CCK-8) assay to examine cell proliferation. The transfected cells were seeded into a 96-well plate at a density of 1000 cells/well. We measured cell viability through the CCK-8 system (Beyotime, China). For EdU assay, 97H and G2 cells were stained using a EdU assay kit (RiboBio, China) following to its instructions. After that, a fluorescence microscope was used to take pictures, with 3 fields randomly selected for each slide. Lastly, the number of EdU-positive cells was counted and quantified. Regarding the colony formation experiment, about 1000-2000 cells were seeded in each well of the 6-well plate, allowing cells to grow until visible colonies were formed.

### Transwell migration and wound healing assays

HCC cells were suspended in 250 μ L of serum-free medium and inserted them into upper chamber of a 24-well Transwell plate (Corning, MA, USA), and the lower chamber was injected with culture medium containing 10% FBS. The transwell chamber was paved with Matrigel coating (2 mg/ml) and DMEM for invasion assays and paved without matrigel mix for migration assays. For the wound-healing closure assay, 97H and G2 cells were cultured in 6-well-plates, linear wounds were scratched with a 10 μL pipette tip. The wound-healing closure was observed and taken photographs under Microscope (Nikon Digital Eclipse C1 system; magnification, x10; Nikon Corporation, Tokyo, Japan).

### Cell cycle analysis

97H and G2 cells were fixed overnight at 4° C in 75% ethanol. Next, we washed away the ethanol using PBS, and incubated the cells with PI and RNase A. After incubating them for 30 mins at 37° C, cell cycle was measured by flow cytometry.

## RESULTS

### Identification of DElncRNAs, DEcircRNAs and DEmRNAs

**Figure 1:**
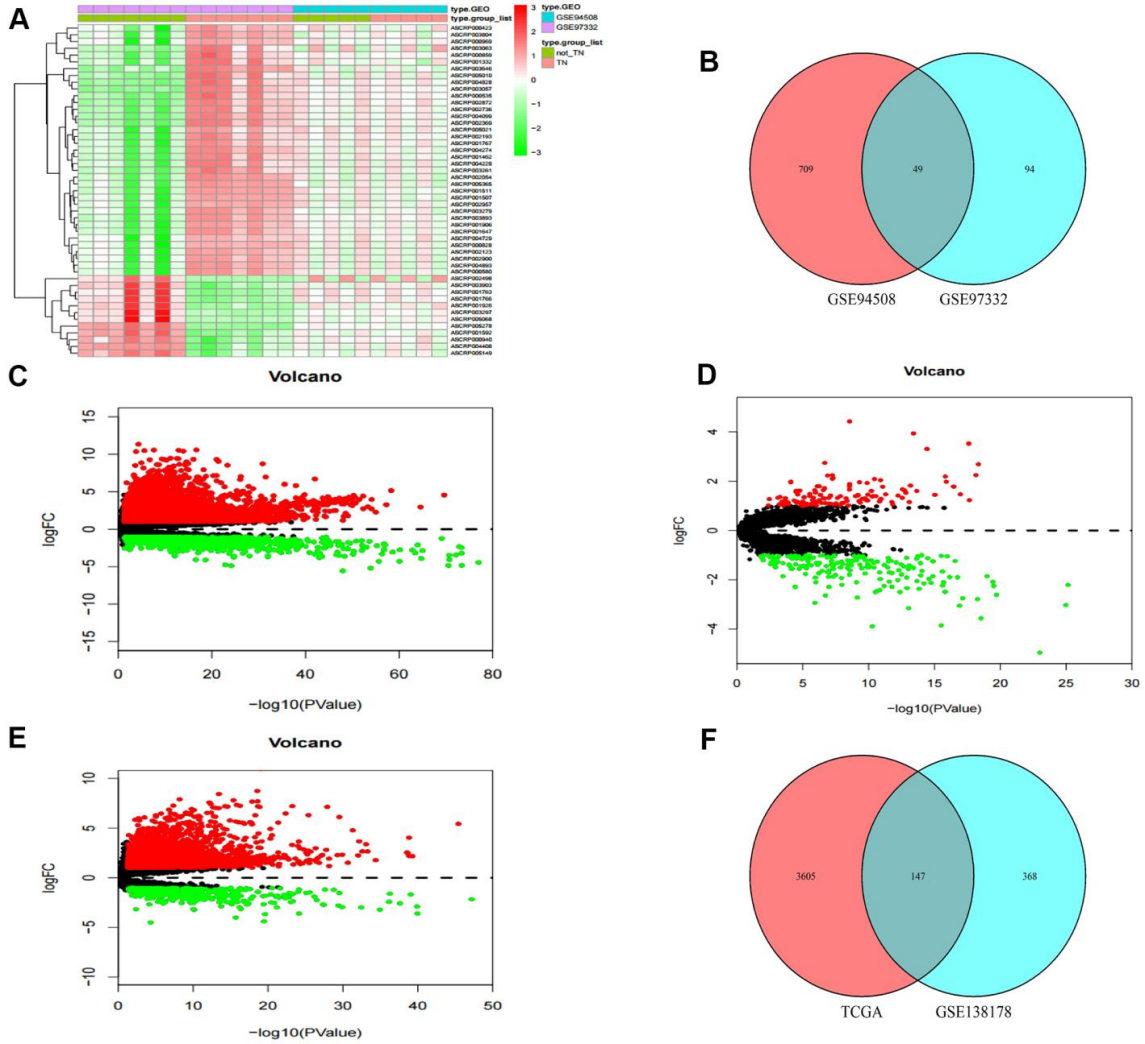
**Identification of differential genes.** (**A**) Heatmap of DEcircRNAs from GEO databases. (**B**) Venn diagram of the intersection of DEcircRNAs. (**C**) Volcano maps of DEmRNAs from TCGA. (**D**) Volcano maps of DElncRNAs from GSE138178. (**E**) Volcano maps of DElncRNAs from TCGA. (**F**) Venn diagram of the intersection of DElncRNAs.

Two circRNA microarray datasets (GSE94508 and GSE97332) were analysed, and the DEcircRNAs between HCC tumor samples and adjacent normal tissues were screened with the criteria |LogFC| > 2 and p < 0.01. We identified 143 DEcircRNAs (93 up-regulated and 50 down-regulated) in the GSE97332 profile, and 758 DEcircRNAs (326 up-regulated and 432 down-regulated) in the GSE94508 profile (Figure 1A). From these two datasets, we screened out a total of 49 overlapping DEcircRNAs for further research (Figure 1B). From the TCGA-LIHC dataset, 5171 DEmRNAs (4064 up-regulated and 1107 down-regulated) were obtained using the criteria |LogFC| > 1 and p < 0.05 (Figure 1C). Then, the same standard was applied to analyze lncRNA data from the GSE138178 dataset and TCGA. A total of 515 DElncRNAs (212 up-regulated and 303 down-regulated) in the GSE138178 profile (Figure 1D), as well as 3752 DElncRNAs (3228 up-regulated and 524 down-regulated) in TCGA (Figure 1E) were identified. Intersection analysis of the two datasets identified 147 DElncRNAs, including 106 with upregulated and 41 with downregulated expression (Figure 1F).

### Prediction of miRNAs targeted by both DElncRNAs and DEcircRNAs

**Figure 2:**
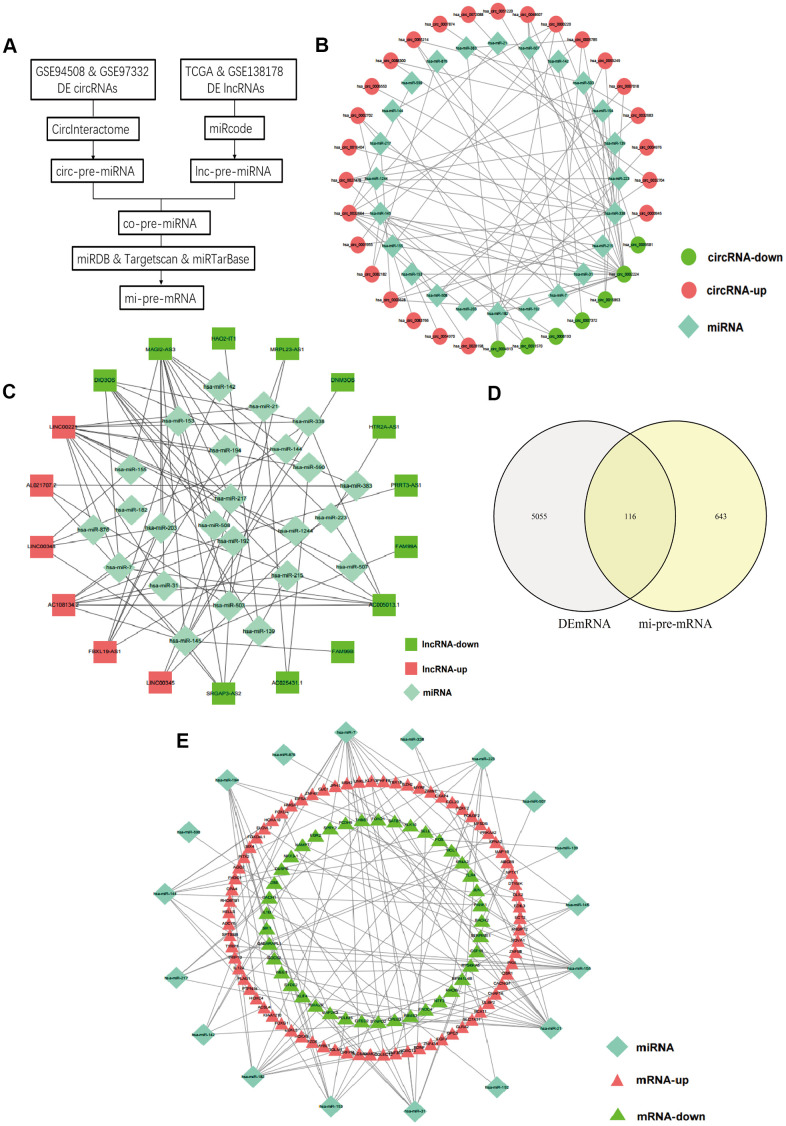
**Prediction of common targeted miRNAs and their targeted DEmRNAs.** (**A**) Flow chart of common pre-miRNAs prediction. (**B**) The relationship between DEcircRNAs and targeted miRNAs. (**C**) The relationship between DElncRNAs and targeted miRNAs. (**D**) Venn diagram of the intersection of circ-pre-miRNAs and lnc-pre-miRNAs. (**E**) The relationship between the common miRNAs and their targeted DEmRNAs.

A flow chart for the creation of common predicted miRNAs was presented in Figure 2A. We predicted the target miRNAs of 147 DElncRNAs through the miRcode database. Then, the interaction between the 147 DElncRNAs and 196 miRNAs was obtained. After searching the CircInteractome database, we identified 49 DEcircRNAs and the targeting 292 miRNAs with mutual interaction ability. The miRNAs targeted by DEcircRNAs and DElncRNAs were crossed to obtain 24 common miRNAs as competitive binding targets, revealing the connection between circRNAs (Figure 2B) and lncRNAs (Figure 2C).

### Prediction of DEmRNAs targeted by miRNAs and screening for hub mRNAs

**Figure 3:**
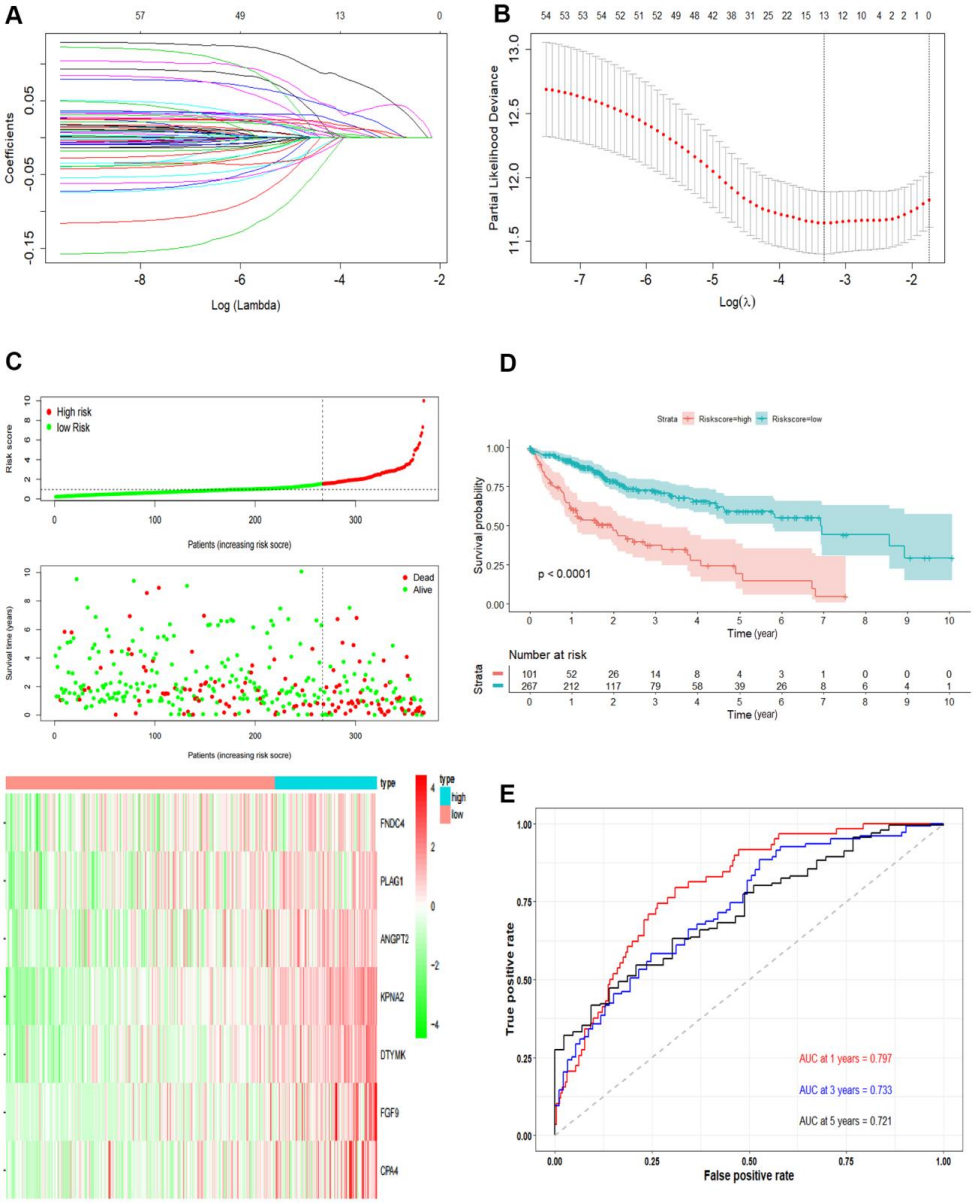
**Development the prognostic model.** (**A**, **B**) Lasso regression analysis results. The trajectory of each independent variable, the horizontal axis represents the log value of the independent variable lambda, and the vertical axis represents the coefficient of the independent variable. The tuning parameter (λ) was calculated based on the partial likelihood deviance with ten-fold cross validation. The dotted vertical lines are drawn at the optimal values by minimum criteria and 1-SE criteria. (**C**) The risk scores distribution, survival status, and gene expression patterns of patients in high and low-risk groups. The dot presents patient status ranked by the increasing risk score. The X axis is patient number and Y axis is survival time. (**D**) Kaplan–Meier survival curve of two groups. (**E**) The time-dependent ROC curves analyses of two groups.

We identified target genes of the common 24 miRNAs by selecting mRNAs shared by miRTarBase, TargetScan, and miRDB database. 759 mRNAs were predicted by all three databases. Then, these candidate target mRNAs and above-mentioned 5171 DEmRNAs were further intersected to obtain 116 hub mRNAs (Figure 2D). Figure 3 presents the relationship between 116 hub mRNAs and 24 common miRNAs (Figure 2E).

### Construction of the prognostic model

Univariate Cox regression was performed and a total of 57 OS-related genes were identified. Then, lasso-penalized Cox analysis was used to further narrow the hub mRNAs (Figure 3A) and 13 hub mRNAs were identified (Figure 3B). After stepwise multivariate Cox regression analysis, 7 mRNAs were finally selected to establish a prognostic model. The seven genes identified were karyopherin subunit alpha 2 (KPNA2), DTYMK, fibroblast growth factor 9 (FGF9), angiopoietin 2 (ANGPT2), PLAG1 zinc finger (PLAG1), fibronectin type III domain containing 4 (FNDC4) and carboxypeptidase A4 (CPA4). The risk score = (0.31119 * expression level of KPNA2) + (0.36809* expression level of DTYMK) + (0.16367 *expression level of FGF9) + (0.16669 * expression level of ANGPT2) + (0.10887 * expression level of PLAG1) + (0.17781 * expression level of FNDC4) + (0.09789 * expression level of CPA4).

Then, we calculated the risk score of each sample based on the seven-gene, and used the *Survminer* R package to find the best cut-off value (Figure 3C). We used the KM curve and ROC curve to assess the prognostic capacity of the seven-gene signature. The KM curves of the two groups were significantly different (P<0.0001; Figure 3D). The prognostic ability of the seven-gene signature can be evaluated based on the ROC curves. The AUCs for 1-year, 3-year, and 5-year OS were 0.797, 0.733, and 0.721, respectively (Figure 3E), indicating that the prognostic model was with high sensitivity and specificity.

### Performance evaluation of the prognostic model

**Figure 4:**
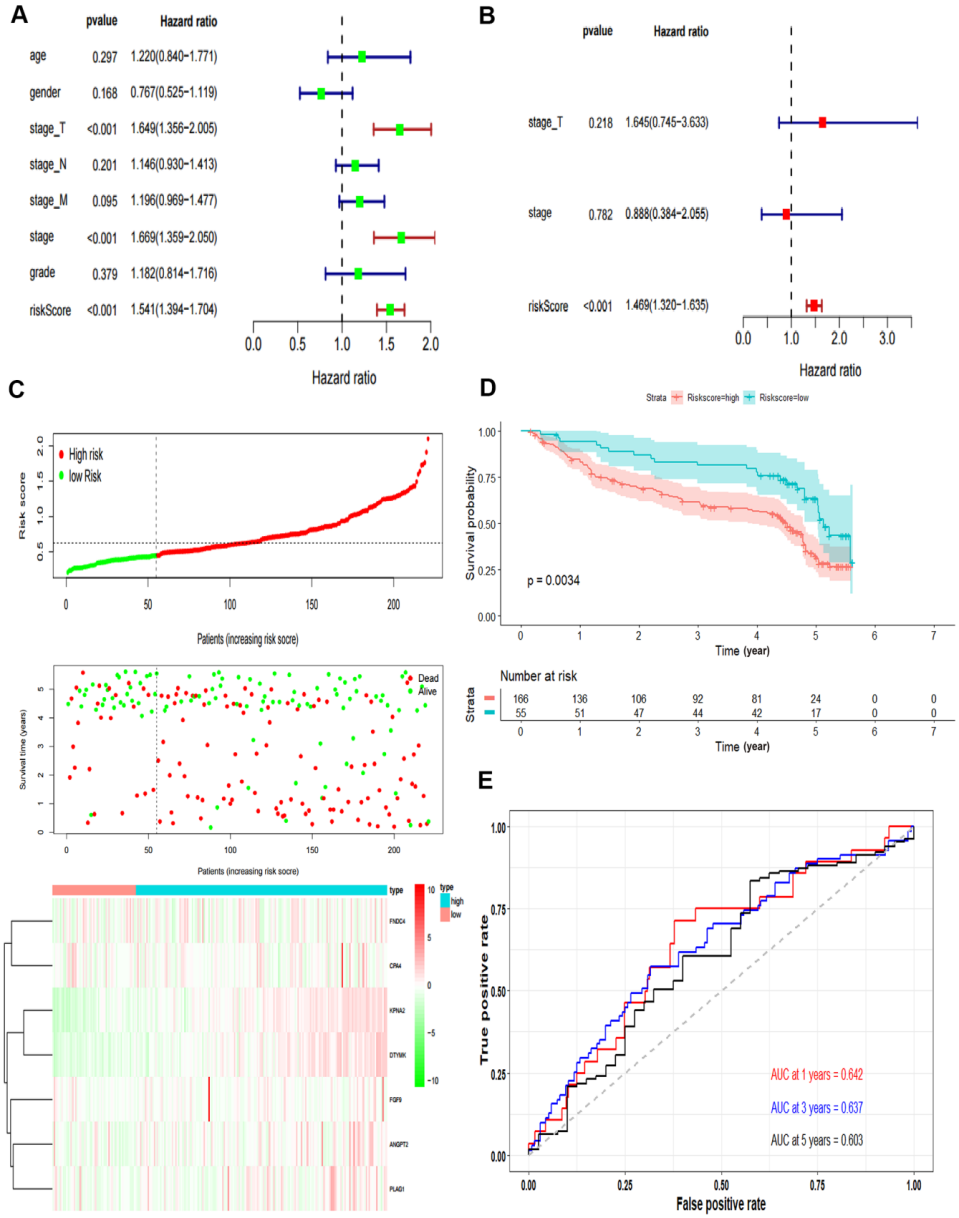
**Validation of the prognostic model.** (**A**) Forrest plot of the univariate Cox regression analysis in TCGA. (**B**) Forrest plot of the multivariate Cox regression analysis in TCGA. (**C**) The risk scores distribution, survival status, and gene expression patterns of HCC patients in GSE14520. The dot presents patient status ranked by the increasing risk score. The X axis is patient number and Y axis is survival time. (**D**) Kaplan–Meier survival curve of two groups in GSE14520. (**E**) The time-dependent ROC curves analyses of two groups in GSE14520.

The independent predictive value of the prognostic model was assessed by the Cox regression analyses in patients from TCGA-LIHC dataset, and the results showed that the pathological stage and risk score had prognostic value (Figure 4A). The risk score of the prognostic model was identified as an independent prognostic factor after multivariate Cox regression analysis (Figure 4B).

In addition, we tested the prognostic model for patients in GSE14520. According to the optimal cut-off value, the dataset was divided into high-risk and low-risk groups (Figure 4C). The OS of patients in the high-risk group was significantly poorer than that of the patients in the low-risk group, which was consistent with the results of the TCGA cohort (P = 0.0034, Figure 4D). The AUCs of the risk score for the 1-year, 3-year and 5-year OS prediction were 0.642, 0.637 and 0.603, respectively (Figure 4E).

### Gene set enrichment analysis

**Figure 5:**
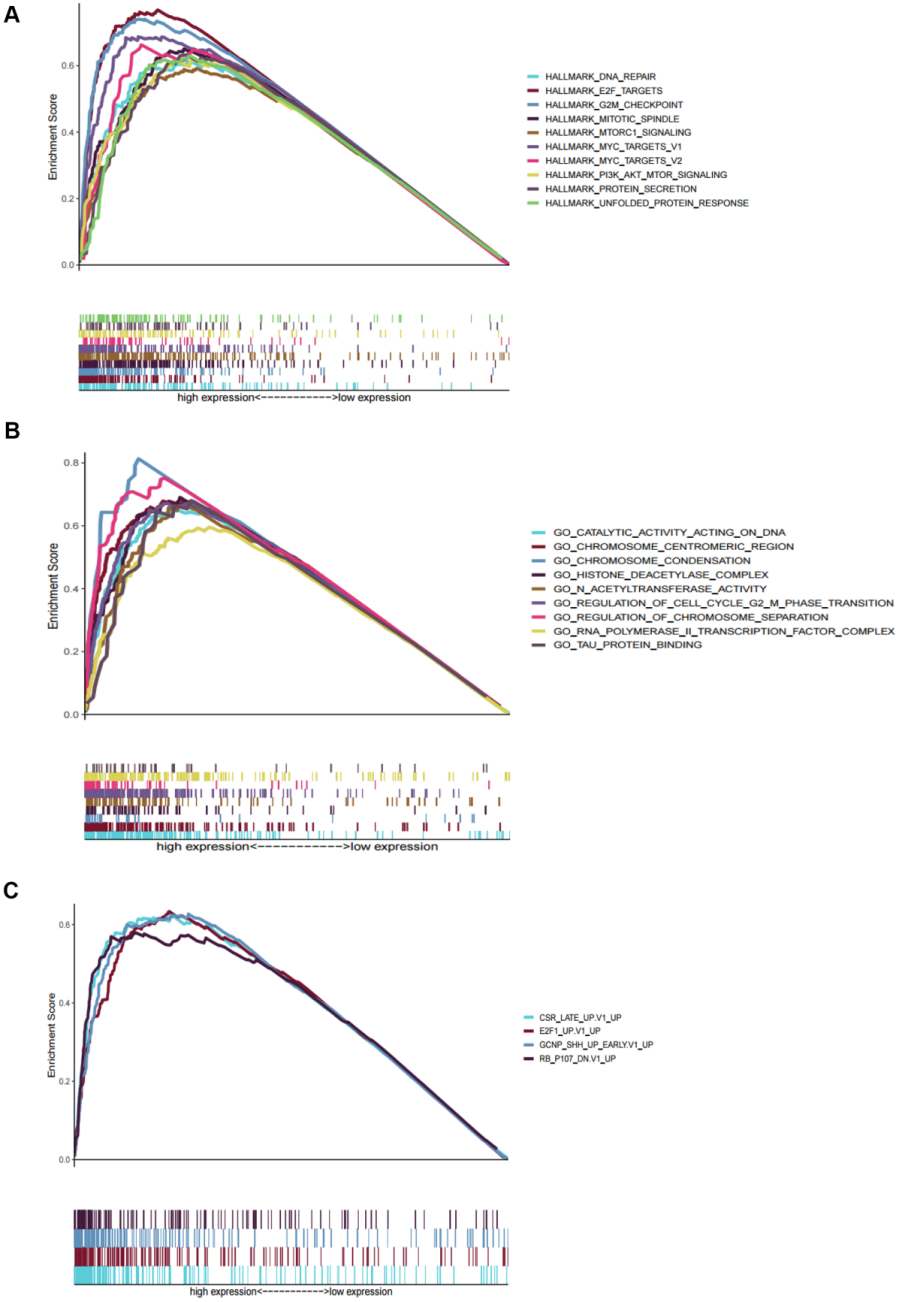
**Gene set enrichment analyses between high and low risk group in TCGA.** (**A**) The top ten significantly enriched cancer hallmarks in high-risk group. (**B**) The significantly enriched GO terms in high-risk group. (**C**) The significantly enriched oncological signatures in high-risk group.

We performed GSEA to analyze HCC patients data in TCGA-LIHC dataset in order to explore the molecular mechanism of the prognostic signature. The results of HCC hallmarks indicated that the high-risk group was significantly enriched in 13 terms, mainly involved in cell cycle, metabolism process (glycolysis, protein secretion), P53 pathway, DNA repair, PI3K/AKT/mTOR signaling so on (Figure 5A). In the high-risk group with prognostic signature, several GO terms were significantly enriched, mainly involved in metabolism process, RNA transport, DNA repair, cell cycle, nucleosome assembly, and protein modification (Figure 5B), which were consistent with the hallmarks analysis results. Besides, oncogenic signatures analysis indicated that 4 oncological signatures including granule cell neuron precursors (GCNP), Rb-P107, E2F Transcription Factor 1 (E2F1), and Early serum response (CSR) were enriched in high-risk group (Figure 5C).

### Bioinformatics analysis of DTYMK in HCC

**Figure 6:**
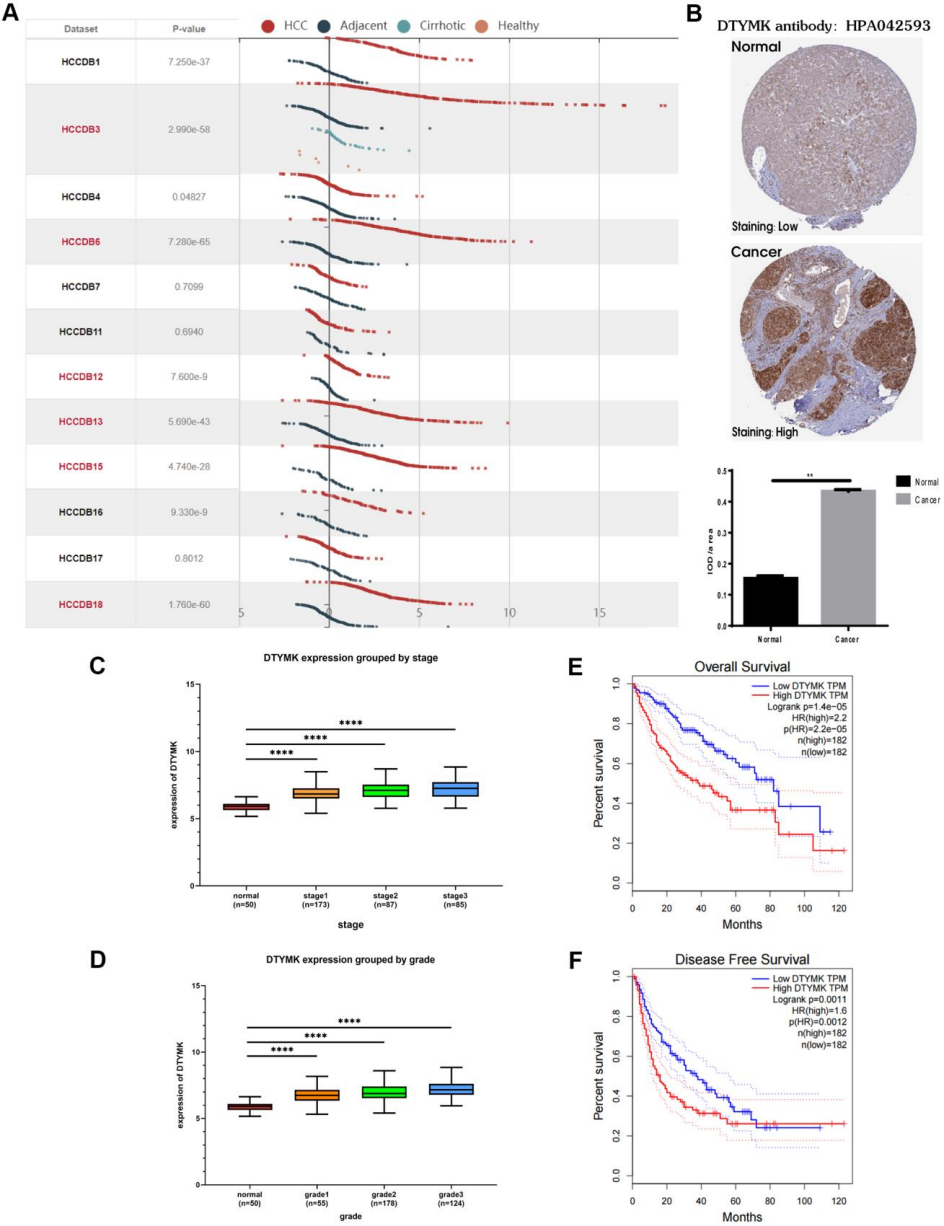
**Bioinformatics analysis of DTYMK in HCC.** (**A**) Gene expression profiles of DTYMK in the HCCDB database. (**B**) Representative immunohistochemistry (IHC) images from the HPA with the DTYMK antibody. (**C**) The expression level of DTYMK was positively correlated with tumor stage in HCC patients. (**D**) The expression level of DTYMK was positively correlated with tumor grade in HCC patients. (**E**) Overall survival analysis of DTYMK in GEPIA. (**F**) Disease free survival analysis of DTYMK in GEPIA. *** represents p < 0.001, ** represents P < 0.01.

In the HCCDB, the expression level of DTYMK in HCC was much higher than that in corresponding paracancerous tissue in most datasets (9/12) (Figure 6A). By examining the DTYMK protein level in HPA, we found that the immunohistochemical staining of HCC tissue was also higher than that of adjacent normal tissues (Figure 6B). Next, we analyzed the correlation between DTYMK mRNA expression and clinicopathological parameters in TCGA. Tumor stage (Figure 6C) and tumor grade (Figure 6D) were found to be highly correlated with the mRNA expression level of DTYMK in HCC patients, indicating high DTYMK expression probably associated with poor clinical characteristics. Then we found that high DTYMK expression is significantly associated with worse OS (Figure 6E, p = 1.4e-05) and RFS (Figure 6F, p = 0.0011) in HCC patients. Finally, the KEGG pathway analysis showed that significant genes differentially expressed related to DTYMK were mainly enriched in base excision repair, purine metabolism, pyrimidine metabolism, spliceosome and DNA replication (Supplementary Table 1).

### Validation of the expression and function of the hub gene DTYMK

**Figure 7:**
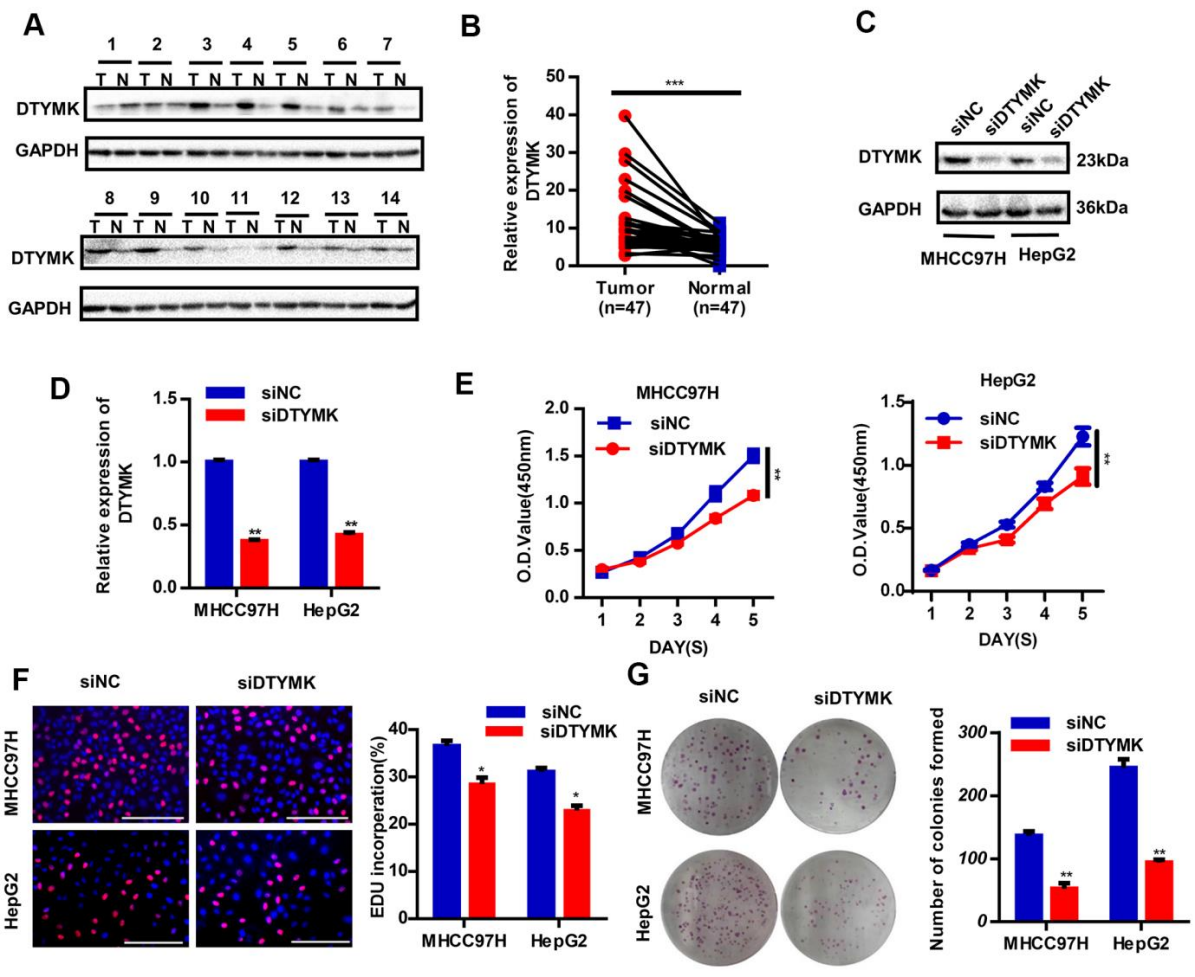
**Validation of DTYMK’s expression and function.** (**A**) The expression levels of DTYMK in HCC and adjacent noncancer tissues were evaluated by Western blot (n=47). (**B**) Statistical analysis of relative DTYMK levels in HCC tissues compared to normal tissue controls (n= 47). (**C**, **D**) Transfection efficiency was verified after transfection of siDTYMK or negative control siRNA. (**E**) HCC cell viability was evaluated with CCK-8 assays. (**F**) EdU assay showed change of proliferative rate after transfection with siDTYMK. (**G**) The number of HCC cell colonies was reduced after transfection with siDTYMK. *** represents p < 0.001, ** represents P < 0.01, * represents P < 0.05.

**Figure 8:**
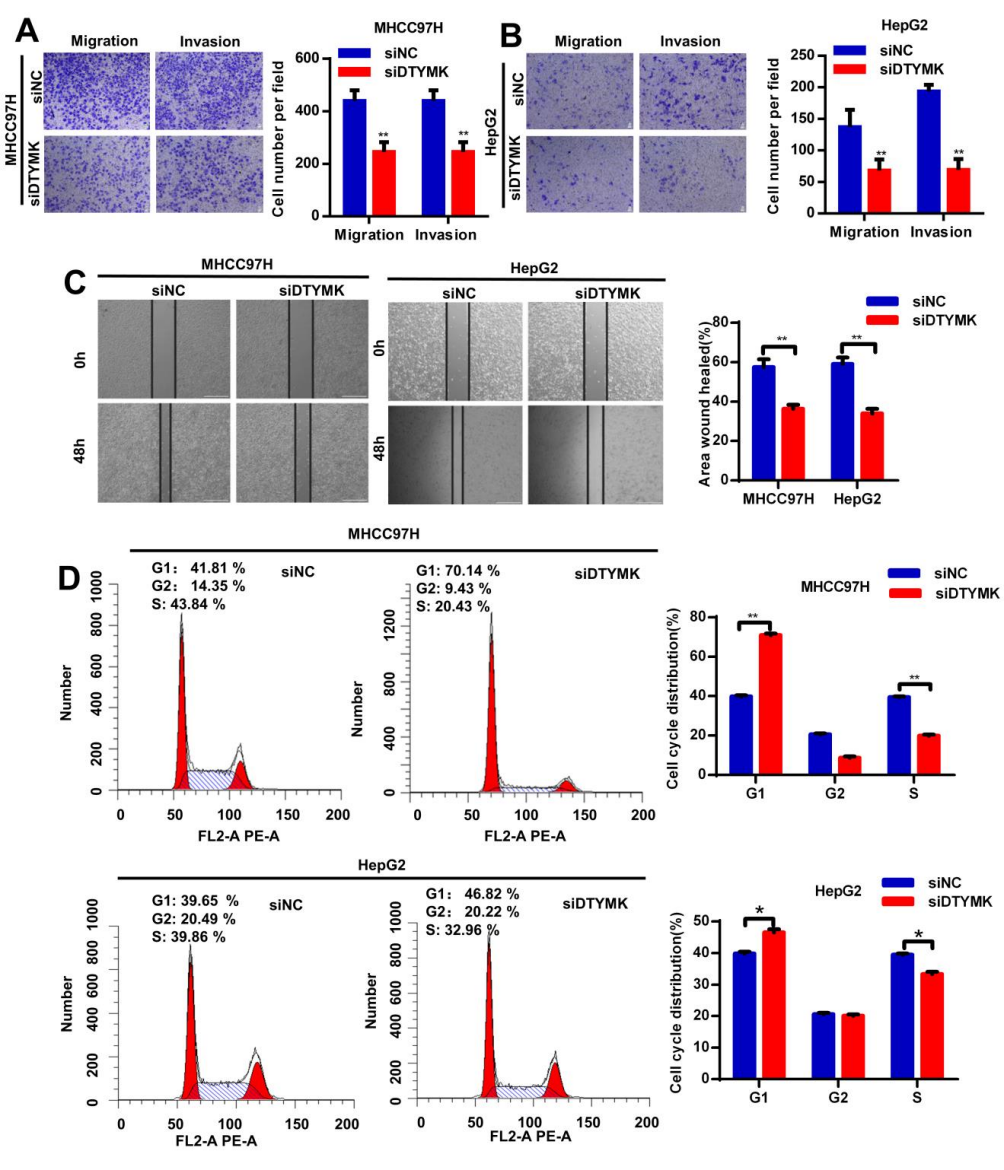
(**A**, **B**) Transwell assays were used to detect HCC cells invasion and migration. (**C**) Effects of DTYMK knockdown on HCC cell migration, as evaluated by wound healing assay. (**D**) Cell cycle was arrested in G0/G1 phase after transfection with siDTYMK in HCC cells. *** represents p < 0.001, ** represents P < 0.01, * represents P < 0.05.

To confirm the *in silico* results, we measured the expression of DTYMK in HCC specimens. It was found that DTYMK was upregulated in HCC specimens in the Tongji cohort (Figure 7A, 7B and Supplementary Figure 1). We then confirmed the knockdown levels of DTYMK by western blot and PCR analysis (Figure 7C, 7D). As expected, CCK-8 assays demonstrated that silencing DTYMK significantly decreased the proliferation of 97H and G2 cells (Figure 7D). Moreover, the EdU assay also suggested a decrease of proliferation ability in 97H and G2 cells after DTYMK silencing (Figure 7E). Furthermore, interference with DTYMK expression inhibited colony formation (Figure 7G), while the results of transwell experiments showed that the migration and invasion rates of 97H and G2 cells transfected with siRNA were significantly lower than that of the control group (Figure 8A, 8B). Accordingly, silencing DTYMK also significantly suppressed wound healing in 97H and G2 cells (Figure 8C). After DTYMK silencing, the proportions of 97H and G2 cells in the G0/G1 phase increased, while that in S-phase significantly decreased, indicating widespread cell cycle arrest (Figure 8D).

### Construction of the prognostic circRNA-lncRNA-miRNA-mRNA ceRNA network in HCC

**Figure 9:**
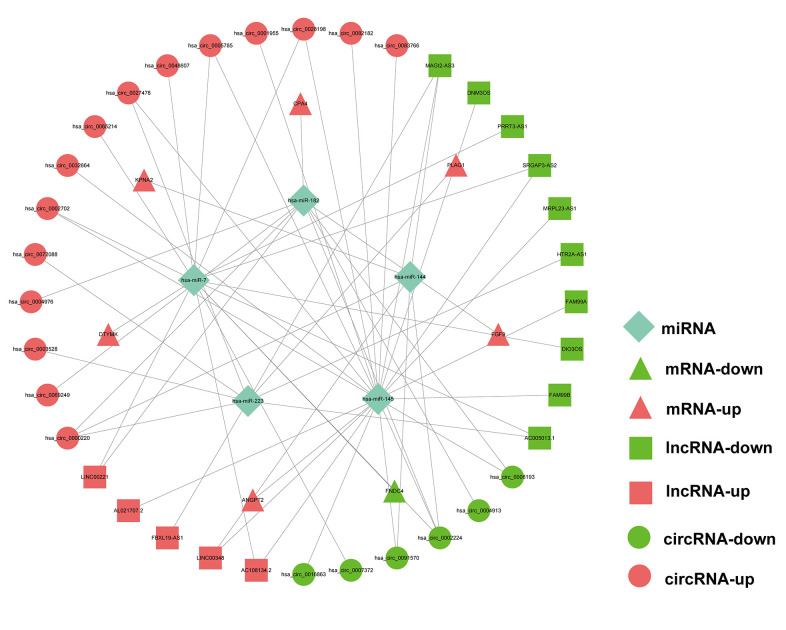
Prognostic circRNA-lncRNA-miRNA-mRNA ceRNA Network in HCC.

As the above-mentioned relationships in the ceRNA network, it was predicted that the 7 hub mRNAs of the prognostic model can interact with 5 miRNAs, which could in turn interact with 21 circRNAs and 15 lncRNAs. Finally, a prognostic circRNA-lncRNA-miRNA-mRNA ceRNA network containing 7 mRNAs, 5 miRNAs, 21 circRNAs and 15 lncRNAs was constructed (Figure 9).

## DISCUSSION

HCC is a refractory disease with high morbidity and mortality worldwide. Although surgical resection can improve the prognosis of some HCC patients, there are still many patients who cannot tolerate current treatments [2]. Moreover, the small number of useful biomarkers makes it challenging to diagnose HCC at an early stage and predict therapeutic effects [5]. Therefore, exploring the biology and novel prognostic biomarkers of HCC may provide clinicians new tools to treat the disease.

The hypothesis of a ceRNA network postulates that circRNAs, lncRNAs, mRNAs, and other types of RNAs compete for binding to miRNAs by sharing MREs, thereby regulating each other's expression and affecting tumorigenesis and cancer progression [23]. The ceRNA regulatory network can better explain the interaction among a variety of RNA types at the genetic level. Some researches have explored and discussed the role of ceRNAs in tumor prognosis and pathogenesis of several cancers. The lncRNA CRNDE acting as a miR-181a-5p sponge was found to regulate the progression and chemoresistance of colorectal cancer through modulating the activity of Wnt/β-catenin signaling [24]. The circular RNA ZFR regulates PTEN through sponge miR-130a/miR-107, which can inhibit the proliferation of gastric cancer cells, induce cell cycle arrest and promote cell apoptosis [25]. LncRNA GAS6-AS2 promotes the proliferation and metastasis of bladder cancer cells with a mechanism in which GAS6-AS2 may function as a ceRNA by directly sponging miR-298 to regulate the CDK9 expression [26]. Bai et al. constructed a ceRNA network composed of lncRNAs, miRNAs, and mRNAs according to their mutual targeting relationships, and used the lncRNAs of the ceRNA network to build a prognostic lncRNA model for HCC [4]. In another study, Wang et al. established a ceRNA network based on 6 circRNAs, 11 miRNAs, and 114 mRNAs to explore a circRNA signature related to HCC [27]. However, these studies only explored the role of either lncRNA-miRNA-mRNA or circRNA-miRNA-mRNA networks in HCC, and there has been no comprehensive simultaneous screening of circRNAs, lncRNAs, miRNAs, and mRNAs to construct a prognostic ceRNA network for HCC. In this study, we comprehensively analyzed the transcriptome data in the TCGA and GEO databases and established the prognostic circRNA-lncRNA-miRNA-mRNA ceRNA network of HCC.

In addition, 116 hub mRNAs were screened, after which Lasso, univariate, and multivariate Cox analyses were performed to construct a prognostic model for HCC. The seven genes KPNA2, DTYMK, FGF9, ANGPT2, PLAG1, FNDC4 and CPA4 were finally selected to construct the prognostic model. The AUC values of the prognostic model for predicting the 1, 3 and 5-year survival were 0.797, 0.733 and 0.721, respectively, indicating that the signature had a good performance in survival prediction. Subsequently, we not only proved that the seven-gene signature was an independent prognostic factor for HCC patients, but also verified its survival predictive ability using the external HCC cohort in the GEO database. All these results indicate that the risk model can serve as a useful prognostic predictor for HCC patients. Therefore, the seven-gene signature can provide insights into biological aspects of HCC and may be a useful guide for individualized management of the disease.

Five hub genes in the prognostic signature (KPNA2, PLAG1, FGF9, ANGPT2 and CPA4) are already known to have a role in HCC. KPNA2 has been linked to cancer in many studies, including HCC. In lung adenocarcinoma, patients with elevated KPNA2 expression level had worse prognosis [28]. High KPNA2 expression is positively correlated with tumor differentiation, vascular invasion, and staging in HCC [29]. Furthermore, it was reported that PLAG1 is a candidate oncogene and could be a critical mediator of the effects of KPNA2 in malignant diseases. A recent study proved that KPNA2 plays an essential role in the nuclear import of PLAG1 and could be a prognostic predictor for HCC patients [30]. It has been reported that FGF9 can promote the carcinogenicity and sorafenib resistance of HCC cells, and the overexpression of FGF9 is related to the poor prognosis of HCC patients [31]. ANGPT2 has been found to have an important influence on angiogenesis and therapy resistance. Recent studies indicated that exosomal ANGPT2 secreted by HCC cells can induce tumor angiogenesis via a novel pathway that is different from the classic ANGPT2/Tie2 pathway, and blocking ANGPT2 is a promising therapeutic strategy for HCC [32]. CPA4, a member of the metallo-carboxypeptidase family, is overexpressed in a variety of cancers. It has been implicated that CPA4 leads to a poor prognosis by regulating tumor proliferation and the expression of stem cell characteristics, and can be used as a potential therapeutic target for HCC patients [33]. The biological functions in HCC of the two other hub genes identified in the signature, FNDC4 and DTYMK, have not yet been elucidated. We finally chose DTYMK for deeper exploration. DTYMK is a nuclear-encoded deoxythymidylate kinase, which can catalyze the phosphorylation of deoxy TMP to deoxy TDP. In the HCCDB and HPA database, we detected that DTYMK was overexpressed in cancerous tissues at both the protein and mRNA levels. Additionally, DTYMK expression correlated with histologic grade, and tumor stage. We subsequently conducted Kaplan-Meier analysis in the TCGA-LIHC cohort and found that patients with high DTYMK expression in cancerous tissues had shorter OS and RFS. Therefore, DTYMK can be a potential clinical biomarker for HCC.

The GSEA results presented that the significantly differentially expressed genes related to DTYMK are mainly enriched in base excision repair, purine metabolism, pyrimidine metabolism, spliceosome and DNA replication, indicating that DTYMK may affect the occurrence of HCC. In addition, we have verified the role of DTYMK in hepatoma cell lines, and the results were consistent with the conclusions of the *in silico* analysis of data in the public databases.

The most of the 15 hub lncRNAs in the prognostic ceRNA network are relevant for various cancers, such as lung adenocarcinoma, breast cancer, colorectal cancer, and especially HCC. The results showed that lncRNA MAGI2-AS3 can recruit KDM1A and promote demethylation of RACGAP1 promoter to prevent the development of HCC [34]. The lncRNA DIO3OS was shown to prevent the development of HCC by disrupting the Hedgehog pathway and sponging miR-328 [35]. Among the circRNAs in the prognostic ceRNA network, hsa_circ_0005785, hsa_circ_0091570 and hsa_circ_0072088 were shown to have an important influence in the biological processes of HCC [36–38]. In summary, the prognostic ceRNA network identified in this study not only contains a series of ncRNAs with unequivocal functions in HCC but also potential unexplored ncRNAs requiring deeper exploration.

However, current research still has some limitations. No *in vivo* and further *in vitro* experiments were performed to verify the function of DTYMK in HCC. Additionally, the role of some hub ncRNAs in the prognostic ceRNA network should be verified in future experiments.

## CONCLUSIONS

A prognostic circRNA-lncRNA-miRNA-mRNA ceRNA network for HCC was constructed for the first time, and a seven-gene signature was identified and validated in TCGA and GEO. One hub gene in the prognostic signature, DTYMK, was identified as a novel potential biomarker for HCC through data mining and experiments.

## Supplementary Material

Supplementary Figure 1

Supplementary Table 1
