# Supplementary material for: Comprehensive analysis of the competing endogenous circRNA-lncRNA-miRNA-mRNA network and identification of a novel potential biomarker for hepatocellular carcinoma
Source: Aging (Albany NY). 2021 May 28;13(12):15990–6008. doi: 10.18632/aging.203056 (PMC8266324; doi:10.18632/aging.203056)
Supplement: Supplementary Figure 1 [file aging-13-203056-s001.pdf]

SUPPLEMENTARY FIGURE

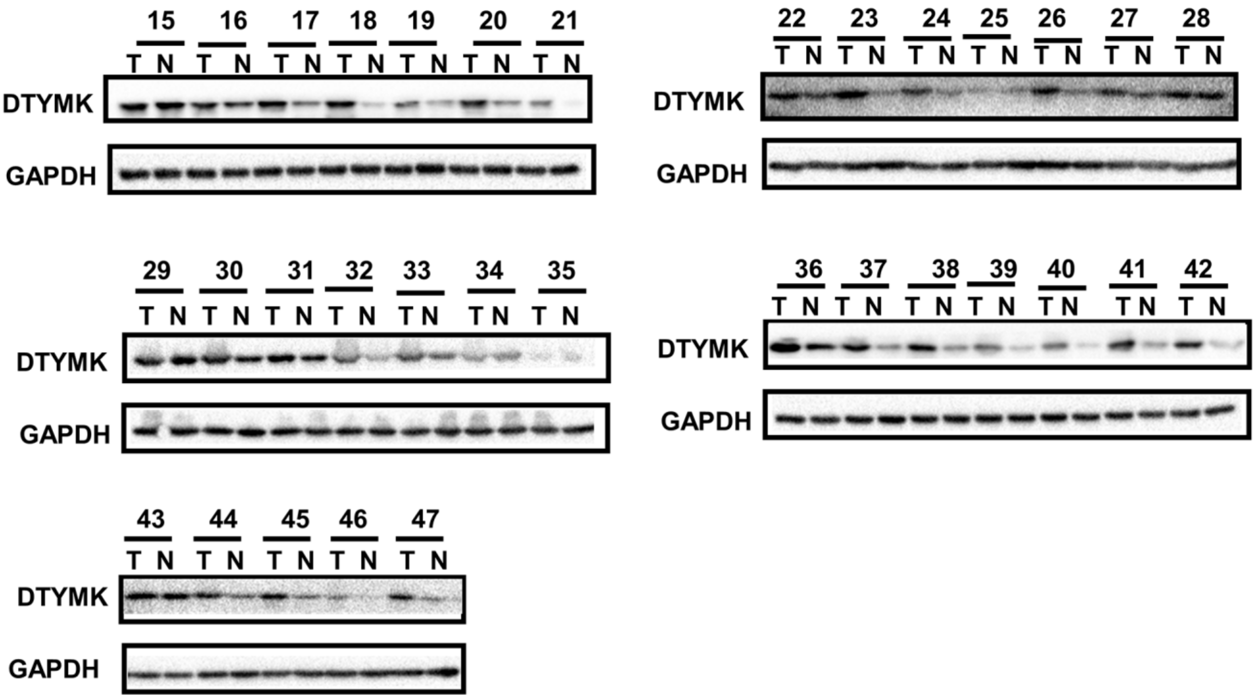

Supplementary Figure 1. The expression levels of DTYMK in HCC and adjacent noncancer tissues were evaluated by western blot.
