# Supplementary material for: Comprehensive analysis of the competing endogenous circRNA-lncRNA-miRNA-mRNA network and identification of a novel potential biomarker for hepatocellular carcinoma
Source: Aging (Albany NY). 2021 May 28;13(12):15990–6008. doi: 10.18632/aging.203056 (PMC8266324; doi:10.18632/aging.203056)
Supplement: Supplementary Table 1 [file aging-13-203056-s002.pdf]

SUPPLEMENTARY TABLE

Supplementary Table 1. Gene set enrichment analyses of DTYMK.

Terms enriched in low risk group					
**NAME**	**SIZE**	**ES**	**NES**	**NOM p-val**	**FDR q-val**
**C2 KEGG (24 terms)**					
KEGG_SPLICEOSOME	126	0.775081	2.135403	0	0.001998
KEGG_DNA_REPLICATION	36	0.860379	2.12395	0	0.00212
KEGG_PYRIMIDINE_METABOLISM	98	0.662451	2.165589	0	0.002664
KEGG_PURINE_METABOLISM	155	0.584109	2.170445	0	0.002771
KEGG_CELL_CYCLE	124	0.691622	2.101745	0	0.002836
KEGG_HOMOLOGOUS_RECOMBINATION	28	0.793349	2.077105	0	0.003134
KEGG_BASE_EXCISION_REPAIR	33	0.795473	2.175527	0	0.005543
KEGG_OOCYTE_MEIOSIS	112	0.596855	2.028667	0	0.007171
KEGG_PROGESTERONE_MEDIATED_OOCYTE_MATURATION	85	0.593121	2.003857	0	0.010455
KEGG_PROTEASOME	44	0.762751	1.98	0	0.012702
KEGG_NUCLEOTIDE_EXCISION_REPAIR	44	0.707281	1.965285	0	0.015007
KEGG_RNA_DEGRADATION	55	0.678917	1.953	0	0.016233
KEGG_MISMATCH_REPAIR	23	0.781214	1.942946	0	0.017833
KEGG_VIBRIO_CHOLERAE_INFECTION	54	0.578596	1.914598	0	0.02124
KEGG_N_GLYCAN_BIOSYNTHESIS	46	0.646307	1.916532	0.003984	0.022681
KEGG_RNA_POLYMERASE	29	0.709808	1.892586	0	0.023505
KEGG_SNARE_INTERACTIONS_IN_VESICULAR_TRANSPORT	38	0.645813	1.892655	0	0.024914
KEGG_UBIQUITIN_MEDIATED_PROTEOLYSIS	132	0.602973	1.871809	0	0.028213
KEGG_HUNTINGTONS_DISEASE	166	0.56755	1.86452	0.008016	0.028414
KEGG_AMINO_SUGAR_AND_NUCLEOTIDE_SUGAR_METABOLISM	43	0.585295	1.850774	0.003922	0.031407
KEGG_RIG_I_LIKE_RECEPTOR_SIGNALING_PATHWAY	70	0.529559	1.856896	0.003953	0.031773
KEGG_BASAL_TRANSCRIPTION_FACTORS	35	0.625064	1.826115	0.005988	0.03617
KEGG_LYSOSOME	121	0.543795	1.829145	0.003945	0.036872
KEGG_ENDOCYTOSIS	181	0.508121	1.798864	0.001883	0.045734
